# Prevalence and related factors of dyslipidemia among urban adults aged 35 to 79 years in Southwestern China

**DOI:** 10.1038/s41598-021-96864-w

**Published:** 2021-09-02

**Authors:** Chuan Huang, Wen-Qiang Zhang, Wei-Wei Tang, Ya Liu, Jian-Xiong Liu, Rong-Hua Xu, Shui-Ping Zhao, Tzung-Dau Wang, Xiao-Bo Huang

**Affiliations:** 1 Department of Cardiology, the Second People’s Hospital of Chengdu, Chengdu, Sichuan China; 2 Department of Epidemiology and Health Statistics, West China School of Public Health and West China Fourth Hospital, Sichuan University, Chengdu, Sichuan China; 3 School of Health Policy and Management, Nanjing Medical University, Nanjing, Jiangsu Province, China; 4 Center for Global Health, Nanjing Medical University, Jiangsu Province, Nanjing, China; 5 Department of Endocrinology and Metabolism, Second People’s Hospital of Chengdu, Chengdu, Sichuan China; 6 Stroke Center, Second People’s Hospital of Chengdu, Chengdu, Sichuan China; 7 Department of Cardiology, Cent S Univ, Xiangya Hosp 2, Changsha, Hunan Province China; 8 Division of Cardiology, Department of Internal Medicine, National Taiwan University Hospital, Taipei City, Taiwan

**Keywords:** Dyslipidaemias, Risk factors, Epidemiology

## Abstract

This study aimed to investigate the prevalence of dyslipidemia and its related factors among urban adults aged 35 to 79 years in Southwestern China. From September 2013 to March 2014, a multi-stage sampling was conducted, and a total of 10,221 people aged 35–79 years living in Chengdu and Chongqing were included. More than 30 investigators were trained in data collection, including questionnaire, anthropometric measurements and blood biomarkers testing. The prevalence of high triglycerides (≥ 2.3 mmol/L), high total cholesterol (≥ 6.2 mmol/L), high low-density lipoprotein cholesterol (≥ 4.1 mmol/L), low high-density lipoprotein cholesterol (< 1.0 mmol/L), and dyslipidemia were 15.7% (95% confidence interval, 15.0–16.4%), 5.4% (4.9–5.8%), 2.5% (2.2–2.8%), 5.7% (5.3–6.2%), and 27.4% (26.5–28.2%), respectively. The prevalence of dyslipidemia was positively correlated with higher education level, monthly income over 2000 CNY, smoking, hypertension, diabetes, overweight and obesity, and central obesity, and negatively correlated with daily physical exercise. The prevalence of dyslipidemia in Southwestern China is lower than the national average level, with high triglycerides being the most common form of dyslipidemia.

## Introduction

Arteriosclerotic cardiovascular disease (ASCVD) is the leading cause of death in both developed countries^1,2^ and most developing countries, including China^3^. The 2007 overall death rate^2^ from CVD was 251.2 per 100,000 in the United States. Dyslipidemia is one of the most important risk factors for ASCVD^4^, leading to atherosclerosis^5^ and increased morbidity and mortality from coronary heart disease^6^ and ischemic stroke^7^. With the rapid economic development, the prevalence of dyslipidemia in Chinese adults has been increasing^6,8–10^. Chengdu and Chongqing, the Chengdu-Chongqing economic circle, are representative metropolitans with soaring economic progress of Southwestern China. There has been scarce epidemiological evidence regarding dyslipidemia in Southwestern China. We conducted a community-based cross-sectional survey to assess the burden of dyslipidemia among urban adults in Southwestern China and provide advice on dyslipidemia management.

## Results

### Demographic and clinical characteristics of the study participants

Among the 10,221 respondents (Table 1), 3474 were men and 6747 were women. The mean age was 55.0 ± 10.7 years, and men had a higher average age. Men had higher prevalence of monthly income over 2000 CNY, smoking, drinking, and daily exercise. The 2 h blood glucose, triglycerides, total cholesterol (TC), high-density lipoprotein cholesterol (HDL-C), and low-density lipoprotein cholesterol (LDL-C) levels were lower in men, and there was no significant difference in fasting blood glucose levels. Men had higher waist circumference (WC), systolic blood pressure (SBP), and diastolic blood pressure (DBP) levels, and lower BMI levels than women. The prevalence of hypertension in men was slightly higher than that in women, and there was no significant difference in the prevalence of diabetes.

**Table 1:** Demographic and clinical characteristics of study participants.

Variables	Total (*n* = 10,221)	Male (*n* = 3474)	Female (*n* = 6747)	*P*
Age (years), mean (SD)	55.0 (10.7)	56.2 (10.9)	54.3 (10.5)	< 0.001
Married (%)	9321 (91.2)	3311 (95.3)	6010 (89.1)	< 0.001
High school education or above (%)	2424 (23.7)	1116 (32.1)	1308 (19.4)	< 0.001
Monthly income (≥ 2000 CNY) (%)	1925 (18.8)	831 (23.9)	1094 (16.2)	< 0.001
Current cigarette smoking (%)	2288 (22.4)	2082 (59.9)	206 (3.1)	< 0.001
Alcohol drinking (%)	205 (2.0)	199 (5.7)	6 (0.1)	< 0.001
Regular physical exercise (%)	428 (4.2)	194 (5.6)	234 (3.5)	< 0.001
Hypertension (%)	3780 (37.0)	1348 (38.8)	2432 (36.0)	0.006
Diabetes mellitus (%)	2111 (20.7)	701 (20.2)	1410 (20.9)	0.395
BMI (kg/m^2^), mean (SD)	23.9 (3.5)	23.6 (3.2)	24.0 (3.6)	< 0.001
WC (cm), mean (SD)	81.1 (10.4)	82.6 (10.3)	80.3 (10.4)	< 0.001
SBP (mmHg), mean (SD)	130.8 (21.2)	132.7 (19.9)	129.8 (21.8)	< 0.001
DBP (mmHg), mean (SD)	78.4 (11.3)	80.4 (11.2)	77.4 (11.2)	< 0.001
Fast glucose (mmol/L), mean (SD)	5.7 (1.8)	5.7 (1.9)	5.6 (1.7)	0.239
2-h plasma glucose (mmol/L), mean (SD)	7.9 (3.8)	7.7 (3.8)	8.0 (3.8)	< 0.001
Triglyceride (mmol/L), median (interquartile range)	1.3 (0.9–1.9)	1.2 (0.9–1.9)	1.3 (0.9–1.9)	0.010
Total cholesterol (mmol/L), mean (SD)	4.6 (0.9)	4.5 (0.9)	4.7 (0.9)	< 0.001
HDL cholesterol (mmol/L), mean (SD)	1.4 (0.4)	1.3 (0.4)	1.4 (0.3)	< 0.001
LDL cholesterol (mmol/L), mean (SD)	2.5 (0.8)	2.5 (0.8)	2.6 (0.8)	< 0.001

BMI, body mass index; WC, waist circumference; SBP, systolic blood pressure; DBP, diastolic blood pressure.

### Age-specific prevalence of dyslipidemia

**Figure 1:**
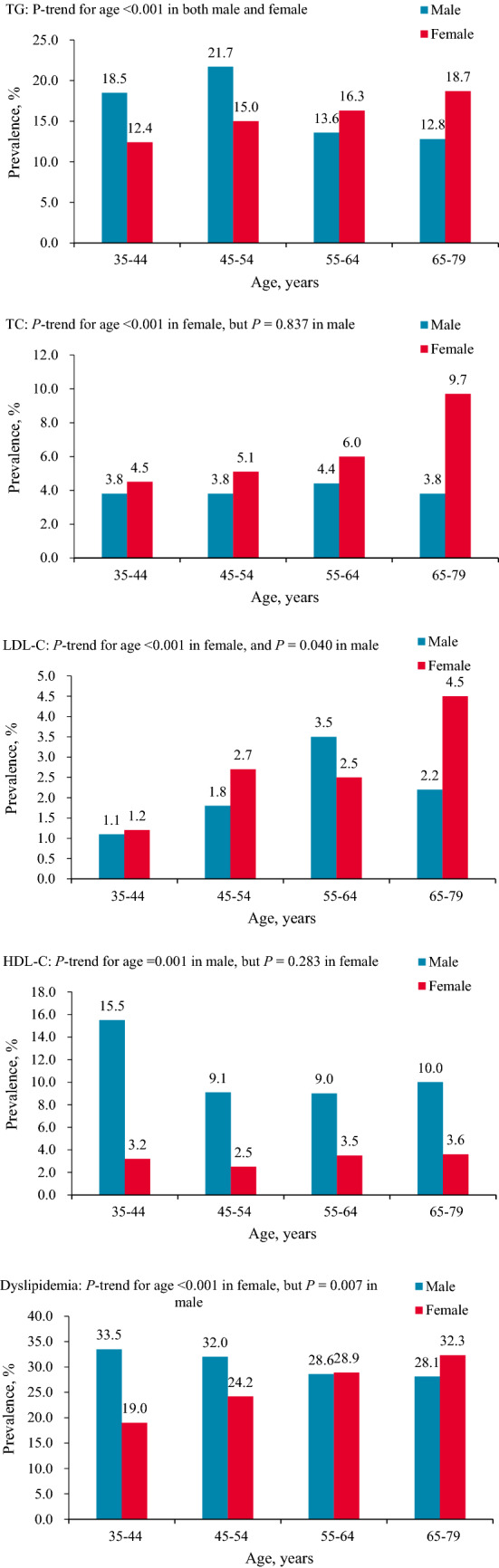
Age-specific prevalence of high triglycerides, high total cholesterol, high low-density lipoprotein cholesterol, low high-density lipoprotein cholesterol, and dyslipidemia among adults aged ≥ 35 years in Southwestern China.

Figure 1 shows the relationships between age, sex, and the prevalence of specific types of dyslipidemia. High TG: In men, compared with those of 35–44 years, it reached the highest in participants of 45–54 years, and then decreased in those of 55–64 and 65–79 years. In women, the prevalence of high TG increased with aging. High TC: In men, there was no significant change with age, while in women, the prevalence of high TC increased with aging. High LDL-C: In men, the prevalence increased with aging, reaching the highest in participants of 55–64 years, and then decreased. In women, the prevalence fluctuated with age. Low HDL-C: In men, the prevalence was highest in participants of 35–44 years and then declined. Among women, there was no significant change. Dyslipidemia: Prevalence decreased with aging in men and increased with aging in women.

### Sex- and age-specific mean of lipid levels

Table 2 shows the lipid levels in subgroups stratified by sex and ages. In men, triglycerides levels showed a downward trend with aging, while LDL-C and HDL-C levels showed an upward trend. There was no significant change in TC level across ages. In women, triglycerides, TC, and LDL-C levels showed an upward trend, while HDL-C levels showed no significant trend.

**Table 2:** Sex- and age-specific mean (95% confidence interval) of serum total cholesterol, HDL cholesterol, LDL cholesterol, and triglycerides levels among adults aged ≥ 35 years in Southwestern China.

Variables	TG†(mmol/L)	TC(mmol/L)	LDL-C(mmol/L)	HDL-C(mmol/L)
**Sex- and age-specific**				
*Male, age (years)*				
35–44	1.40 (1.34–1.46)	4.48 (4.42–4.55)	2.40 (2.34–2.45)	1.28 (1.26–1.30)
45–54	1.47 (1.41–1.54)	4.52 (4.46–4.58)	2.43 (2.38–2.49)	1.40 (1.36–1.44)
55–64	1.28 (1.24–1.32)	4.48 (4.43–4.53)	2.48 (2.43–2.52)	1.35 (1.33–1.36)
65–79	1.22 (1.17–1.26)	4.52 (4.46–4.58)	2.56 (2.51–2.62)	1.35 (1.33–1.38)
*P* value for linear trend‡	< 0.001	0.669	< 0.001	0.013
**Female, age (years)**				
35–44	1.24 (1.20–1.27)	4.48 (4.44–4.53)	2.36 (2.33–2.40)	1.40 (1.38–1.41)
45–54	1.31 (1.28–1.34)	4.69 (4.65–4.73)	2.55 (2.51–2.58)	1.50 (1.48–1.52)
55–64	1.44 (1.41–1.47)	4.81 (4.77–4.85)	2.66 (2.63–2.70)	1.45 (1.44–1.47)
65–79	1.50 (1.45–1.55)	4.87 (4.82–4.93)	2.66 (2.61–2.71)	1.42 (1.40–1.43)
*P* value for linear trend‡	< 0.001	< 0.001	< 0.001	0.491

TG, triglyceride; TC, total cholesterol; LDL-C, low density lipoprotein cholesterol; HDL-C, high density lipoprotein cholesterol.

^†^Serum triglyceride measurements have a right-skewed distribution, so geometric mean and 95% confidence interval are calculated.

^‡^*P* value from linear regression.

### Prevalence of dyslipidemia

Table 3 shows the relationships between different factors and the prevalence of dyslipidemia. The prevalence of high TG, high TC, high LDL-C, low HDL-C, and dyslipidemia were 15.7% (95% confidence interval, 15.0–16.4%), 5.4% (4.9–5.8%), 2.5% (2.2–2.8%), 5.7% (5.3–6.2%), and 27.4% (26.5–28.2%), respectively. The prevalence of dyslipidemia increased with aging, and was higher in men (30.2%) than in women (25.9%). Elevated TG was related to daily exercise, hypertension, diabetes, overweight, and central obesity. TC increased with aging, gender, marital status, education level, smoking status, high blood pressures, diabetes, overweight, and central obesity. Elevated LDL-C was associated with aging and diabetes. Lower HDL-C was associated with gender, education level, income, smoking status, high blood pressures, overweight, and central obesity. Dyslipidemia was associated with aging, sex, education, monthly income, smoking status, daily exercise, hypertension, diabetes, overweight, and central obesity.

**Table 3:** Prevalence of dyslipidemia by selected characteristics.

Variables	Characteristics	High TG	High TC	High LDL-C	Low HDL-C	Dyslipidemia
Age (years)	35–44	322 (14.3)	97 (4.3)	27 (1.2)	160 (7.1)	531 (23.6)
	45–54	436 (16.8)	122 (4.7)	63 (2.4)	112 (4.3)	683 (26.3)
	55–64	510 (15.4)	181 (5.5)	94 (2.8)	180 (5.4)	955 (28.8)
	65–79	333 (16.2)	148 (7.2)	73 (3.6)	130 (6.3)	627 (30.5)
	*P*_*Trend*_	0.264	< 0.001	< 0.001	0.544	< 0.001
Gender	Female	1043 (15.5)	409 (6.1)	176 (2.6)	214 (3.2)	1748 (25.9)
	Male	558 (16.1)	139 (4.0)	81 (2.3)	368 (10.6)	1048 (30.2)
	*P*	0.427	< 0.001	0.397	< 0.001	< 0.001
Married	No	135 (15.0)	64 (7.1)	27 (3.0)	40 (4.4)	230 (25.6)
	Yes	1466 (15.7)	484 (5.2)	230 (2.5)	542 (5.8)	2566 (27.5)
	*P*	0.566	0.015	0.330	0.090	0.205
High school education or above	No	1203 (15.4)	466 (6.0)	201 (2.6)	383 (4.9)	2062 (26.4)
	Yes	398 (16.4)	82 (3.4)	56 (2.3)	199 (8.2)	734 (30.3)
	*P*	0.241	< 0.001	0.462	< 0.001	< 0.001
Monthly income (≥ 2000 CNY)	No	1286 (15.5)	456 (5.5)	205 (2.5)	425 (5.1)	2179 (26.3)
	Yes	315 (16.4)	92 (4.8)	52 (2.7)	157 (8.2)	617 (32.1)
	*P*	0.348	0.208	0.561	< 0.001	< 0.001
Current cigarette smoking	No	1215 (15.3)	462 (5.8)	212 (2.7)	331 (4.2)	2092 (26.4)
	Yes	386 (16.9)	86 (3.8)	45 (2.0)	251 (11.0)	704 (30.8)
	*P*	0.071	< 0.001	0.058	< 0.001	< 0.001
Alcohol drinking	No	1566 (15.6)	540 (5.4)	253 (2.5)	567 (5.7)	2738 (27.3)
	Yes	35 (17.1)	8 (3.9)	4 (2.0)	15 (7.3)	58 (28.3)
	*P*	0.575	0.349	0.603	0.311	0.761
Regular physical exercise	No	1567 (16.0)	526 (5.4)	251 (2.6)	557 (5.7)	2709 (27.7)
	Yes	34 (7.9)	22 (5.1)	6 (1.4)	25 (5.8)	87 (20.3)
	*P*	< 0.001	0.835	0.133	0.893	0.001
Hypertension	No	828 (12.9)	302 (4.7)	157 (2.4)	328 (5.1)	1483 (23.0)
	Yes	773 (20.4)	246 (6.5)	100 (2.6)	254 (6.7)	1313 (34.7)
	*P*	< 0.001	< 0.001	0.517	0.001	< 0.001
Diabetes mellitus	No	1055 (13.0)	363 (4.5)	176 (2.2)	445 (5.5)	1953 (24.1)
	Yes	546 (25.9)	185 (8.8)	81 (3.8)	137 (6.5)	843 (39.9)
	*P*	< 0.001	< 0.001	< 0.001	0.077	< 0.001
Overweight or obesity	No	563 (10.1)	266 (4.8)	146 (2.6)	231 (4.2)	1133 (20.4)
	Yes	1038 (22.3)	282 (6.0)	111 (2.4)	351 (7.5)	1663 (35.7)
	*P*	< 0.001	0.005	0.430	< 0.001	< 0.001
Central obesity	No	818 (11.5)	357 (5.0)	177 (2.5)	334 (4.7)	1611 (22.7)
	Yes	783 (25.0)	191 (6.1)	80 (2.6)	248 (7.9)	1185 (37.8)
	*P*	< 0.001	0.028	0.864	< 0.001	< 0.001

### Multivariable-adjusted *OR*s and 95%*CI* for dyslipidemia

Table 4 shows the results of multivariable logistic regression analysis. High TG was positively correlated with high school education, smoking, hypertension, diabetes, overweight and obesity, central obesity, but negatively correlated with daily physical exercise. Compared with participants of 35–44 years, TG elevation was higher in participants of 45–54 years but lower in those of 65–79 years. For high TC, there was a positive correlation with diabetes, and a negative correlation for women with education levels of high school or above. Compared with participants of 35–44 years, those of 65–79 years had a higher risk of TC elevation. Elevated LDL-C was positively correlated with age and diabetes. Decreased HDL-C was positively correlated with female, high school education, monthly income over 2000 CNY, smoking, overweight and obesity, and central obesity but negatively correlated with aging (35–44 years of age as a reference). Dyslipidemia was positively correlated with high school education, monthly income over 2000 CNY, smoking, hypertension, diabetes, overweight and obesity, central obesity, but negatively correlated with daily physical exercise.

**Table 4:** Multivariable–adjusted *OR*s and 95% *CI* for different definitions of dyslipidemia among adults aged 35 years or older in Southwestern China.

Variables	Characteristics	High TG	High TC	High LDL-C	Low HDL-C	Dyslipidemia
Age (years)	35–44	1	1	1	1	–
	45–54	1.19 (1.01–1.39)	1.10 (0.83–1.44)	2.05(1.30–3.24)	0.60 (0.47–0.78)	–
	55–64	0.88 (0.75–1.03)	1.13 (0.87–1.47)	2.27 (1.47–3.50)	0.71 (0.56–0.89)	–
	65–79	0.76 (0.64–0.91)	1.46 (1.12–1.92)	2.70 (1.72–4.24)	0.74 (0.58–0.95)	–
Gender	Female	–	1	–	1	–
	Male	–	0.67 (0.55–0.82)	–	2.80 (2.23–3.52)	–
High school education or above	No	1	1	–	1	1
	Yes	1.30 (1.14–1.48)	0.64 (0.50–0.82)	–	1.51 (1.24–1.84)	1.40 (1.25–1.56)
Monthly income (≥ 2000 CNY)	No	–	–	–	1	1
	Yes	–	–	–	1.27 (1.03–1.56)	1.23 (1.10–1.38)
Current cigarette smoking	No	1	–	–	1	1
	Yes	1.33 (1.17–1.52)	–	–	1.54 (1.23–1.92)	1.38 (1.24–1.54)
Alcohol drinking	No	–	–	–	–	–
	Yes	–	–	–	–	–
Regular physical exercise	No	1	–	–	–	1
	Yes	0.49 (0.34–0.71)	–	–	–	0.72 (0.56–0.92)
Hypertension	No	1	–	–	–	1
	Yes	1.34 (1.19–1.51)	–	–	–	1.38 (1.26–1.52)
Diabetes mellitus	No	1	1	1	–	1
	Yes	2.03 (1.79–2.30)	1.86 (1.54–2.25)	1.61 (1.22–2.12)	–	1.77 (1.59–1.97)
Overweight or obesity	No	1	–	–	1	1
	Yes	1.76 (1.54–2.01)	–	–	1.65 (1.35–2.03)	1.71 (1.54–1.90)
Central obesity	No	1	–	–	1	1
	Yes	1.78 (1.56–2.03)	–	–	1.66 (1.35–2.04)	1.43 (1.28–1.60)

“–” indicates that an independent variable was not associated with the dependent variable.

## Discussion

This study investigated dyslipidemia and related risk factors in urban adults aged 35–79 years in Chengdu and Chongqing from September 2013 to March 2014, using a multi-stage sampling in Southwestern China. In this study, the prevalence of dyslipidemia was 27.4%, which increased with aging, and was higher in men (30.2%) than in women (25.9%). The higher prevalence in men may be related to the higher income, smoking rate, drinking rate, WC, hypertension, SBP, and DBP levels. The prevalence of dyslipidemia in women was lower than that in men in participants under 55 years of age, whereas the prevalence was higher in women than in men in later life. With the increase of age, TG, TC, and LDL-C levels in women showed an increasing trend. This result was consistent with other studies^9,12^. This may be related to the changes in estrogen levels in women before and after menopause^12^. Our results showed that dyslipidemia was unrelated to age and gender. This may be due to the varied age-related changes in the prevalence of dyslipidemia between men and women.

The national chronic kidney disease survey in China^13^ showed that the prevalence of dyslipidemia in Chinese adults was 34.0%^9^. The prevalence of dyslipidemia in Southwestern China is lower than Chinese adults. Besides, the prevalence of dyslipidemia (27.4%) and average lipid levels (TG 1.3 mmol/L and TC 4.6 mmol/L) in Southwestern China were lower than that in other regions of China as previously reported. For example, the cities with developed economy, such as Beijing^14^ (dyslipidemia 35.4%, TG 1.53 mmol/L, TC 5.05 mmol/L), Shanghai^12^ (dyslipidemia 36.5%), Shenzhen^15^ (dyslipidemia 34.72%, TG 1.44 mmol/L, TC 4.77 mmol/L), Jilin^16^ (dyslipidemia 62.1%) and Shandong^17^ (dyslipidemia 45.8%). The prevalence of dyslipidemia displays obvious economic and regional difference. This may be related to the following reasons: (1) The economically developed regions like Beijing^14^, Shanghai^12^ and Guangzhou^15^ have higher living standards, and their lifestyles and consumptions of Western diet are close to developed countries; (2) The cold climate in the northern region leads to an increased intake of animal fats, a decreased intake of fresh fruits and vegetables, and restriction of outdoor physical activity, thus increasing the risk of obesity and related metabolic abnormalities^16,17^.

Chengdu is the capital of Sichuan Province, compared with the epidemiological survey of dyslipidemia in Sichuan^18^ in 2002, the prevalence of dyslipidemia was increased to a certain extent (22.5% vs 27.4%). The lipid levels were also increased. TG and TC levels were 1.14 and 3.76 mmol/L, respectively, in 2002 and 1.3 and 4.6 mmol/L in this study. These increases are consistent with the rapid economic development and lifestyle changes in the Southwestern region. Although the current prevalence of dyslipidemia and lipid levels in the Southwestern urban people aged 35–79 were still lower than that in the other regions in China, the increasing trends indicate that prevention and management of dyslipidemia in Southwestern China should be seriously implemented.

The prevalence of high TG, high TC, low HDL-C, and high LDL-C in people aged 35–79 years in Southwestern China were 15.7%, 5.4%, 5.7%, and 2.5%, respectively, while the prevalence of high TG, high TC, low HDL-C and high LDL-C in Chinese adults^9^ were 12.2%, 7.5%, 15.3%, and 8.0%, respectively. High TG was the most common dyslipidemia in Southwestern China. The prevalence of high LDL-C was much lower in Southwestern China. Compared to the other lipid parameters, the LDL-C level is most closely related to ASCVD risk^19,20^. A research^21^ involving 219,522 Chinese patients with type 2 diabetes showed that, in Chinese adults with type 2 diabetes, the prevalence of CHD and stroke were lowest in Southwest China. Another national survey^22^ including 480,687 adults aged ≥ 20 years demonstrated that the prevalence of stroke was lowest in Southwestern China. Despite its large population and high GDP, Southwestern China had a lower average number of percutaneous coronary intervention^23^. In contrast to dyslipidemia, recent studies^24,25^ showed that the prevalence of hypertension (38.4%) and diabetes (19.5%) in the Southwestern China was higher than the national average (hypertension 32.5%^26^, diabetes 9.7%^27^). Therefore, it is reasonable to speculate that the lower prevalence of dyslipidemia and lower LDL-C levels contribute to the lower ASCVD prevalence in Southwestern China. Further studies should be conducted to validate our findings.

This study also analyzed the correlations between dyslipidemia and various factors. In addition to the common risk factors associated with dyslipidemia^10,28^, such as smoking, hypertension, and diabetes, this study also showed a positive correlation between a high educational level, a monthly income of more than 2000 CNY, and the prevalence of dyslipidemia. This may be due to the better economic and nutritional status of people with higher education and incomes^29^. Overweight, obesity, and central obesity are correlated with dyslipidemia, suggesting that BMI and waist circumference can be used as screening indicators for dyslipidemia. Weight control is an important prevention and control method^30,31^. Besides, dyslipidemia was negatively correlated with daily physical exercise, suggesting that strengthening physical exercise is also an important method for the prevention and control of dyslipidemia.

This study has several limitations. In the study design, we did not consider clusters of population sampled with varying probabilities according to their size. We excluded about 24% of participants lacking significant information, such as demographic information, anthropometric measurements, and lipid measurements, which may cause potential selection bias. To minimize the potential selection bias, we calculated the associated Clopper-Pearson 95% confidence intervals for prevalence. Participants enrolled by a multi-stage (district-subdistrict-community) sampling were adults in Chengdu and Chongqing, the most advanced cities in Southwestern China. Five selected districts in this study may not well represent urban areas of Southwestern China or primary sampling frames. A mass of the rural population has been migrating to that both cities due to the government’s urbanization policies, and the study population cannot reflect ethnic diversity in Southwestern China. Thus, the sampling error was underestimated in the study population versus the source population. The prevalence observed likely overestimated the prevalence of dyslipidemia among adults in Southwestern China. Of note, large samples can partially compensate loss of accuracy.

In conclusion, our study provides the latest information regarding dyslipidemia in Southwestern China. The results show that the prevalence of dyslipidemia in Southwestern China was lower than the national average. High TG is the main type of dyslipidemia, and the prevention and control of dyslipidemia is challenging. Dyslipidemia is closely related to smoking, hypertension, diabetes, higher education, higher incomes, obesity, and central obesity. Only by strengthening public health education and intervening in risk factors can we deal with the challenge.

## Methods

### Study population

From September 2013 to March 2014, a multi-stage (district-subdistrict-community) sampling was conducted in Chengdu and Chongqing. First, Jinjiang district, Longquanyi district, and Chenghua district were randomly sampled from urban areas in Chengdu, and Yubei district and Jiangbei district were randomly sampled from urban areas in Chongqing. Second, a subdistrict was randomly sampled from each district. Finally, a community was randomly s sampled from each subdistrict, and residents in this community who met the inclusion and exclusion criteria were selected. Among the 14,061 eligible participants from five representative urban communities, 13,378 people aged 35–79 years living in Chengdu and Chongqing were enrolled, yielding a response rate of 95.1% (13,378/14,061). The ethics committee of the Second People’s Hospital of Chengdu approved this study protocol (NO 2013015). All participants provided written informed consent.

### Inclusion and exclusion criteria

From September 2013 to March 2014, residents aged 35 to 79 years who had lived in selected communities for more than five years were included in the study. People with secondary hypertension, mental illness, malignancy, renal failure requiring dialysis, or refusal to participate were excluded. Due to the lack of demographic information and weight, blood pressure, waist circumference, or body mass index (BMI) data, and lipid measurements, 10,221 participants were included in the final analysis.

### Data collection

More than 30 investigators were trained in data collection, including questionnaire, anthropometric measurements and blood biomarkers testing. According to the cardiovascular survey methodology developed by the World Health Organization^11^, subjects filled out the same field questionnaire, including demographic characteristics; lifestyle, personal and family history of the disease; measurements of height, weight, waist circumference, and blood pressures; fasting glucose, triglycerides (TG) and total cholesterol (TC) levels were also included. BMI was calculated by dividing weight in kilograms by height in meters squared, and subjects were asked to go barefoot and wear only light clothing when measuring height and weight. The researchers measured the minimum circumference between the lower edge of the rib and the iliac spine to get a waist measurement. Thirty minutes before the measurement of blood pressures, the subjects were told not to drink coffee, tea, or alcohol, and not to smoke or exercise. The subjects sat for a five-minute break, then had their blood pressures measured while they sat with a mercury sphygmomanometer. Systolic blood pressure (SBP) and diastolic blood pressure (DBP) recorded the first occurrence of Korotkoff sound (stage I) and the disappearance of Korotkoff sound (stage V), respectively, and averaged blood pressure readings of the two measurements. Fasting venous blood was collected. All blood samples were sent to the Clinical Laboratory Center of Second People's Hospital of Chengdu and Clinical Laboratory Center of the Second Affiliated Hospital of Chongqing Medical University. Total cholesterol, TG, and blood glucose were measured by enzymatic method. All anthropometric measurements and blood biomarkers testing were carried out in accordance with relevant guidelines and regulations.

### Index definition

Smoking is defined as having smoked more than 100 cigarettes in a lifetime. Drinking is defined as consuming more than 30 g of alcohol per week for more than a year. Regular physical activity refers to moderate or vigorous activity of 30 min or more on at least 3 days a week^8^. Hypertension is defined as having a definite medical history, and/or systolic blood pressure greater than or equal to 140 mmHg, and/or diastolic blood pressure greater than or equal to 90 mmHg. Diabetes is defined as having a definite medical history, and/or fasting glucose greater than or equal to 7.0 mmol/L, and/or a 2-h glucose tolerance test greater than or equal to 11.1 mmol/L. According to the guidelines for Prevention and Treatment of Dyslipidemia in Chinese Adults (2016 Revision), dyslipidemia is defined as TC ≥ 6.2 mmol/L, low-density lipoprotein cholesterol (LDL-C) ≥ 4.1 mmol/L, high-density lipoprotein cholesterol (HDL-C) < 1.0 mmol/L, and/or TG ≥ 2.3 mmol/L, and/or self-reported history of dyslipidemia^9^.

### Diagnostic criteria

Dyslipidemia was defined as total cholesterol ≥ 6.2 mmol/L, and/or LDL cholesterol ≥ 4.1 mmol/L, and/or HDL cholesterol < 1.0 mmol /L, and/or TG ≥ 2.3 mmol /L, and/or self-reported history of dyslipidemia, following Chinese Guidelines for Prevention and Treatment of Dyslipidemia in Adults (2016 Revision)^9^.

### Statistical analysis

Absolute number (percentage, %) was used to describe the categorical data, and a Chi-Square test was used to compare the difference between different groups. We calculated 95% confidence intervals for prevalence using Clopper–Pearson method. The data subject to or close to the normal distribution were described by means ± standard deviation, and the difference between different groups was compared by Student’s t-test. The data of skewed distribution were described by the median with the interquartile range, and the comparison between different groups was performed by Wilcoxon rank sum test. Trend analysis was done by Chi-Square trend test or linear regression analysis. Both univariable and multivariable analyses were performed using an unconditional Logistic regression model, and the OR value and its 95% confidence interval were calculated. We used a forward-stepwise selection method (Likelihood Ratio, LR) to specify how independent variables were entered into the multivariable logistic regression model. All statistical analyses were performed with SPSS 23.0 software, and *P* < 0.05 was considered statistically significant.
